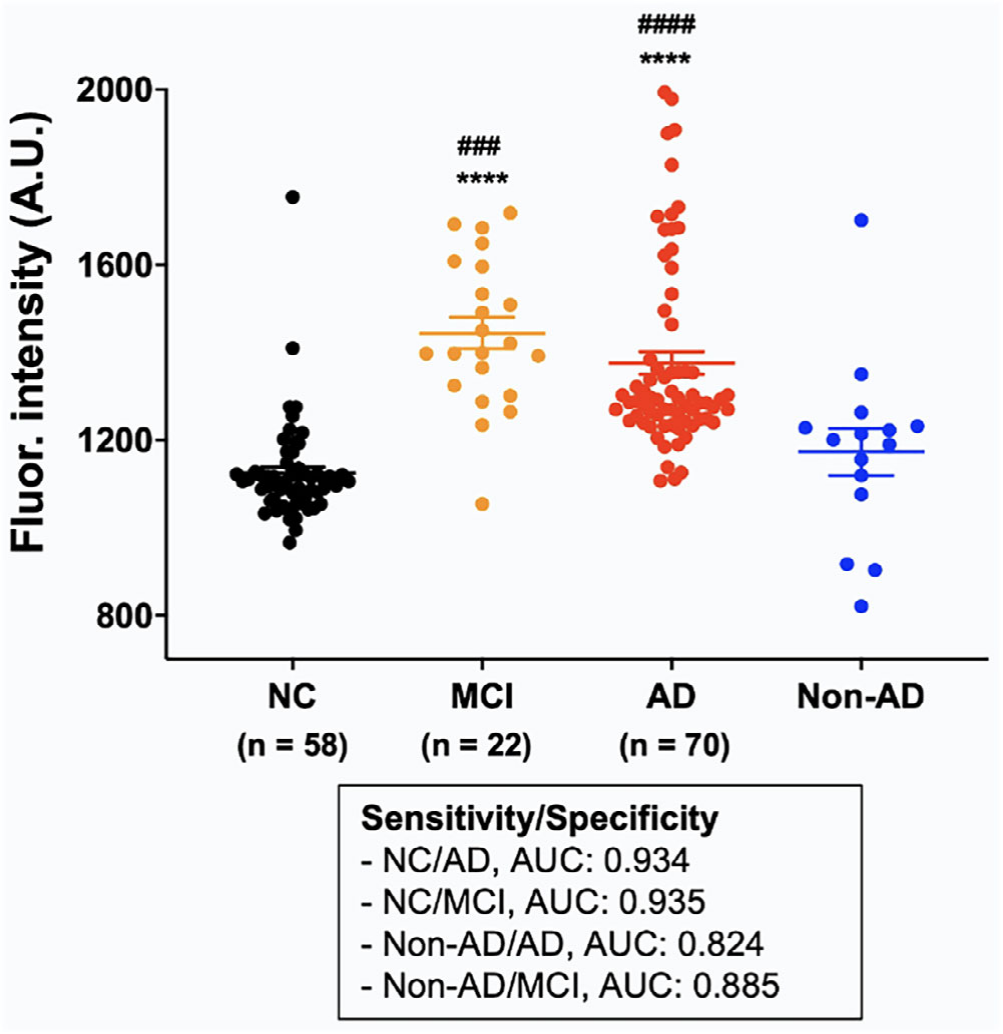# AS‐YFluor, a Novel Fluorescent Imaging Agent Detecting Aβ42 Oligomers in Blood for Alzheimer Diagnosis

**DOI:** 10.1002/alz70856_102424

**Published:** 2025-12-25

**Authors:** YoungSoo Kim, Illhwan Cho, Sunhee Lee, Hye Yun Kim, Ikyon Kim

**Affiliations:** ^1^ Amyloid Solution Inc., Seongnam, Gyeonggi‐do, Korea, Republic of (South); ^2^ College of Pharmacy, Yonsei University, Incheon, Incheon, Korea, Republic of (South)

## Abstract

**Background:**

Amyloid‐β42 (Aβ42) oligomers are neurotoxic aggregates and key biomarkers of Alzheimer's disease (AD), as they are significantly implicated in the early onset and progression of the disorder. To elucidate the disease‐associated roles of Aβ42 oligomers and support AD diagnosis in preclinical stages, Aβ42 oligomer‐imaging probes are under active investigation. However, reliable detection tools for Aβ42 oligomers in clinical trials remain scarce. Herein, we introduce a novel chemical compound, AS‐YFluor, which selectively exhibits heightened fluorescence emission at 475 nm upon binding to Aβ42 oligomers. Utilizing this compound, we measured changes in Aβ42 oligomer levels in the blood plasma of AD patients and cognitively normal controls (NC) to distinguish between groups.

**Method:**

Prior to blood Aβ tests with AS‐YFluor, the selectivity of the compound for Aβ42 oligomers was evaluated through *in vitro* assays. We then examined its diagnostic potential using brain lysate, cerebrospinal fluid, and blood samples from 5XFAD transgenic mice and age‐matched controls. We recruited NC (*n* = 58) and patients with AD (*n* = 70), mild cognitive impairment (MCI) (*n* = 22), and non‐AD dementia (*n* = 15), who underwent assessments including clinical interviews, neurological examinations, neuropsychological and laboratory tests, magnetic resonance imaging, and amyloid–positron emission tomography scans. We added AS‐YFluor to each blood plasma sample and analyzed the fluorescence signal to distinguish each group.

**Result:**

In *in vitro* studies, AS‐YFluor specifically targets oligomeric Aβ42 species, with no fluorescence promotion upon exposure to Aβ monomers, fibrils, Aβ40, pyroglutamate‐modified Aβ, other misfolded proteins, or highly abundant blood proteins such as albumin. The addition of the compound to biological samples from mice facilitated clear differentiation between samples from 5XFAD mice and age‐matched controls. In clinical investigation, AS‐YFluor distinguished AD patients from NC, it also effectively discerned AD patients from non‐AD dementia subjects. Additionally, it demonstrated the ability to indicate AD progression by distinguishing MCI from AD patients and NC with over 93% sensitivity and specificity.

**Conclusion:**

Collectively, AS‐YFluor is a novel fluorescent chemical that selectively detects Aβ42 oligomer levels in the blood of AD patients. Moreover, its versatility extends beyond blood diagnostics and holds promise for application in non‐clinical laboratory settings for oligomer research.